# Explanatory Variables of Objectively Measured 24 h Movement Behaviors in Coronary Heart Disease Patients: A Systematic Review

**DOI:** 10.1002/clc.70440

**Published:** 2026-08-17

**Authors:** Xiaofeng Peng, Ting Guo, Xiuzheng Wang, Yin Wan, Ying Peng, Ting Cheng, Siyue Tang, Hui Tu

**Affiliations:** ^1^ The Second Affiliated Hospital of Nanchang University Nanchang City China; ^2^ Nanchang University School of Nursing Nanchang City China

**Keywords:** 24 h movement behaviors, coronary heart disease, explanatory variables, socioecological model

## Abstract

**Aims:**

This study systematically examined the multilevel determinants influencing 24 h movement behaviors (physical activity, sedentary behavior, and sleep) in coronary heart disease patients on the basis of a socioecological model. The aim was to provide evidence‐based recommendations for behavior modification in coronary heart disease patients.

**Methods:**

We conducted this systematic review adhering to PRISMA 2020. Using the PICOS framework, we included observational studies of adults (≥ 18 years) with coronary heart disease, measuring 24 h movement behaviors (physical activity, sedentary behavior, sleep) objectively. Outcomes included behavioral time allocation and explanatory variables. We systematically searched PubMed, Embase, Web of Science, and the Cochrane Library from inception to November 9, 2025. Data regarding study characteristics, behavioral measurement methods, and explanatory variables were independently extracted. Methodological quality was assessed using the Newcastle−Ottawa Scale for cohort studies and the Joanna Briggs Institute Critical Appraisal Checklist for cross‐sectional studies. The socioecological model served as the analytical framework to organize extracted explanatory variables into four hierarchical levels: intrapersonal, interpersonal, environmental, and policy. This multilevel synthesis provided a systematic architecture for understanding behavioral heterogeneity, moving beyond the simplistic attribution of behaviors to individual physiology. Evidence synthesis and grading were performed using the method proposed by Sallis et al., which entailed calculating the proportion of studies supporting the hypothesized direction of association to evaluate the strength of evidence.

**Results:**

A total of 24 studies were included, although none comprehensively explored 24 h movement behaviors in coronary heart disease patients. Only seven studies explored the determinants of two activity behaviors simultaneously, while the remaining 17 studies focused on isolated behaviors. The findings revealed that the primary explanatory variables were concentrated at the intrapersonal level, with aging significantly associated with reduced physical activity, while participation in cardiac rehabilitation was linked to increased physical activity. At the interpersonal level, social support moderated both physical activity levels and sedentary behavior. At the environmental level, residential characteristics were associated with 24 h movement behavior patterns; however, no significant associations were found at the policy level.

**Conclusion:**

Guided by the socioecological model, this systematic review comprehensively identifies multi‐level explanatory variables of 24 h movement behaviors in coronary heart disease patients. The factors showing the most consistent associations include participation in cardiac rehabilitation, younger age, spousal support, and residential environment. At the intrapersonal level, personalized encouragement to promote patients' participation in cardiac rehabilitation can serve as a core component of behavioral interventions. At the interpersonal and environmental levels, leveraging spousal support and actively optimizing exercise‐related environments in medical facilities and communities are promising directions for intervention.

## Graphical abstract

Applying a socioecological model, this systematic review identifies multi‐level predictors of 24 h movement behaviors in coronary heart disease. Key targets include cardiac rehabilitation, younger age, spousal support, and residential environment, providing evidence for personalized interventions.

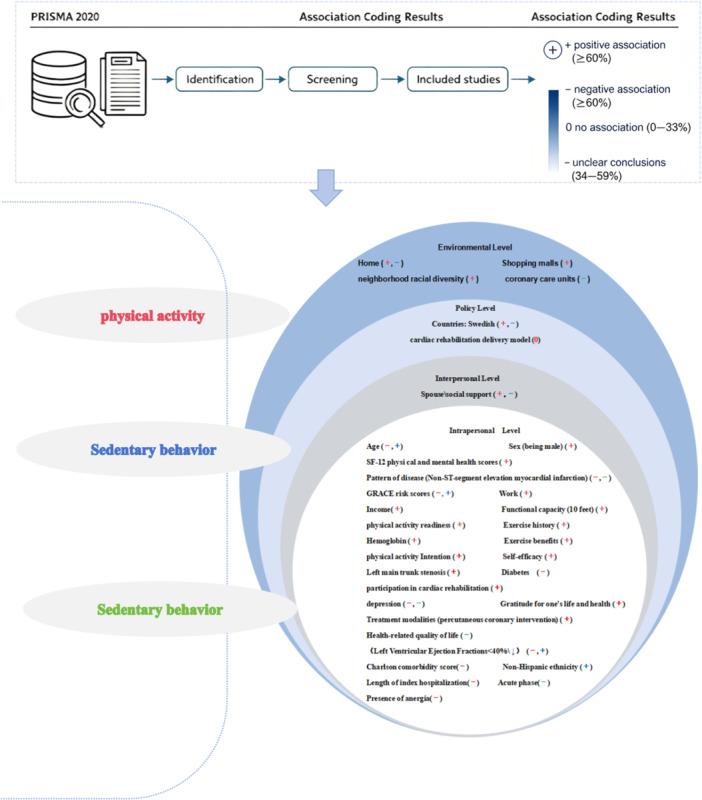

## 1 Introduction

Coronary heart disease represents a significant global health and economic burden. According to statistics, healthcare expenditures related to coronary heart disease reached $1.66 trillion in 2021, and if current trends continue, this cost is projected to increase to $2.59 trillion by 2050. In response to this challenge, the traditional “disease treatment” model is becoming increasingly unsustainable. However, with proactive measures to control and eliminate all behavioral and metabolic risk factors, it is theoretically possible to prevent 82.1% of deaths from atherosclerotic coronary artery disease by 2050, potentially saving approximately 8.7 million lives annually [1]. This immense potential highlights the urgent need to shift coronary heart disease management strategies from the traditional “disease treatment” model to an integrated “secondary prevention priority” approach. In this transition, scientifically integrated regulation of 24 h movement behaviors is regarded as a core strategy. Evidence indicates that combined lifestyle interventions focus not only on changing single behaviors but also on optimizing and substituting the timing of various activities to effectively reduce all‐cause mortality [2]. These findings suggest that future cardiovascular disease prevention and control efforts should focus increasingly on improving overall health outcomes through interventions targeting daily behavior patterns. The term “24 h movement behaviors” refers to the cyclical distribution of physical activity, sedentary behavior, and sleep over a 24 h period. These behaviors interact synergistically to influence overall health [3]. Studies have shown [4] that varying intensities of physical activity, sedentary behavior, and sleep are closely associated with the health status of patients with coronary artery disease. Coronary heart disease patients often display poor health behaviors, such as decreased physical activity, prolonged sedentary behavior, and inadequate or excessive sleep. Therefore, optimizing the distribution of activity behaviors across a 24 h period and further examining the factors that influence these behaviors are essential.

However, the core bottleneck in current clinical practice lies in the excessive homogeneity of existing interventions. Current general intervention protocols are mostly developed based on average population effects and fail to account for the diverse underlying causes of behavioral differences among individual patients, resulting in low clinical implementation rates. Therefore, this systematic review adopts the socioecological model to systematically sort out multi‐level factors influencing health behaviors and explore the potential mechanisms behind the limited effectiveness of existing interventions.

A single biomedical perspective cannot explain why patients with identical clinical characteristics exhibit distinctly different behavioral patterns. The socioecological model provides a systematic framework for understanding behavioral heterogeneity by integrating the interactive effects across four domains: the intrapersonal domain (physical and psychological constraints), the interpersonal domain (family and social support), the environmental domain (community and public facilities), and the policy domain (medical insurance and clinical guidelines). Using this framework, the present review elaborates on the full spectrum of factors associated with 24 h movement behaviors among patients with coronary heart disease. Although this approach cannot directly achieve precise targeting for specific patient subgroups, it identifies the key leverage points at each level that contribute to poor population‐level behavioral outcomes. The findings of this review will lay a solid theoretical foundation and offer clear directions for the development of targeted stratified intervention strategies in future studies.

A systematic analysis of the pathways through which multilevel factors influence behavior can help mitigate the issue of inadequate clinical translation of interventions [5]. Understanding the factors that influence behavior is complex because they are interconnected across various socioecological levels. The most comprehensive and widely used model to summarize these factors is the socioecological model proposed by Sallis et al. [6]. This model comprises (1) the individual level, which includes sociodemographic factors (e.g., age, sex), behavioral factors (e.g., attitudes, beliefs), and health‐related variables (e.g., comorbidities, quality of life); (2) the interpersonal level (e.g., modeling, social support); (3) the environmental level, further divided into physical environment factors (e.g., proximity to green spaces, parks, shops, public transport, and sidewalks); and perceived environmental factors (e.g., community satisfaction, sense of safety); and (4) the policy level (e.g., laws, regulations, educational opportunities, and employment opportunities). Recognizing that behavior change is influenced by multiple levels, this multilevel framework provides the most comprehensive approach to understanding these factors.

The aim of this systematic review is to analyze the factors influencing 24 h movement behaviors in coronary heart disease patients within the socioecological model framework and to provide recommendations for future interventions targeting 24 h movement behaviors in coronary heart disease patients.

## 2 Methods

This systematic review adhered to the Preferred Reporting Items for Systematic Reviews and Meta‐Analyses guidelines [7]. The protocol was registered with PROSPERO (ID:CRD42024583827). A comprehensive search strategy was implemented to systematically identify studies investigating the factors influencing 24 h movement behaviors in patients with coronary heart disease. To minimize recall bias and the potential over‐ or underestimation of results due to self‐reporting, only studies that objectively measured 24 h movement behaviors were included.

### 2.1 Search Strategy

The research question was formulated using the population‐intervention‐outcome framework: “What factors influence objectively measured 24 h activity behaviors in patients with coronary heart disease?” To address this question, three key concepts were identified: coronary heart disease, objective measurement, and activity behaviors. These concepts were linked using the Boolean operators “AND” and “OR.” We systematically searched PubMed, Embase, Web of Science, and the Cochrane Library from inception to November 9, 2025. The search strategy for every database can be found in Supporting Information S1: File 1.

### 2.2 Inclusion and Exclusion Criteria

Inclusion criteria:

1. Diagnosis of coronary heart disease; age 18 years or older.
2. Measurement of 24 h activity behaviors, including moderate‐to‐vigorous physical activity, light physical activity, sedentary behavior, and sleep.
3. At least one of the four factors influencing these behaviors was studied.
4. Full‐text availability.
5. Observational studies, case–control studies, and cohort studies published in English or Chinese.

Exclusion criteria:

1. Animal studies.
2. Nonoriginal research (e.g., letters to authors, conference abstracts, and reviews).
3. Studies using nonobjective data.

Outcome measures:

Changes in the time spent on each activity behavior.

### 2.3 Eligibility Criteria

The study selection process was conducted independently by two authors. First, duplicate records were removed, followed by an eligibility assessment based on the titles and abstracts of the remaining studies. After applying the inclusion and exclusion criteria, the full texts of the eligible studies were reviewed. Additionally, the reference lists of these studies were searched to identify potentially relevant research. In the event of discrepancies during the selection process, a consensus was reached through discussion among three authors.

### 2.4 Data Extraction

Two researchers independently screened the search results and assessed the eligibility of the full‐text reports. Discrepancies were resolved through discussion or by a third researcher. Data were extracted by three independent researchers, and the extracted information included (1) authors and publication year, (2) country, (3) study population, (4) sample size, (5) study design, (6) activity behaviors (i.e., physical activity, sedentary behavior, and/or sleep) and specific behaviors (e.g., light‐intensity physical activity, moderate‐to‐vigorous physical activity), (7) measurement methods, and (8) individual/interpersonal/environmental/policy influencing factors. Detailed information is provided in Supporting Information S1: File 2.

### 2.5 Quality Assessment

Quality assessment was conducted independently by two researchers. For cohort, case–control, and observational studies, the Newcastle–Ottawa Scale was used [8]. The Newcastle–Ottawa Scale evaluates eight criteria across three domains: (1) selection of the study population, (2) comparability between groups, and (3) outcome assessment. The total score ranges from 0 to 9 points. On the basis of previous research [9], studies were classified into the following quality levels: low quality (0–3 points), moderate quality (4–6 points), and high quality (≥ 7 points). For cross‐sectional studies, the Joanna Briggs Institute Critical Appraisal Checklist for Analytical Cross‐Sectional Studies [10] was used. This checklist includes eight questions concerning inclusion criteria, measurement validity and reliability, confounding factors, and statistical analysis, with responses categorized as “Yes,” “No,” “Unclear,” or “Not Applicable.” On the basis of previous research [11], the following scoring was applied on the basis of the yes score: low risk of bias (≥ 70%), moderate risk of bias (50%–69%), and high risk of bias (≤ 49%). Detailed information is provided in Supporting Information S1: File 3.

### 2.6 Data Synthesis

Using the socioecological model, the explanatory variables for 24 h activity behaviors were categorized into four domains: (1) individual level, (2) interpersonal level, (3) environmental level, and (4) policy level. Furthermore, the strength of evidence for the associations between these factors and physical activity, sedentary behavior, and/or sleep was coded on the basis of models used by researchers such as Sallis et al. [12] and Hinkley et al. [13, 14]. The method for calculating the proportion of associations supporting the hypothesized direction was as follows: the number of results supporting the association (e.g., positive correlation) divided by the total number of results mentioning the association (including positive, negative, and no correlation). The association coding framework was defined as follows: “0” indicated no association (0%–33% of studies support the hypothesized association), “?” indicated unclear conclusions (34%–59% of studies supported the association), and “+” or “–” indicated a positive or negative correlation (≥ 60% of studies supported the direction of the relationship).

## 3 Results

### 3.1 Study Characteristics

**Figure 1:**
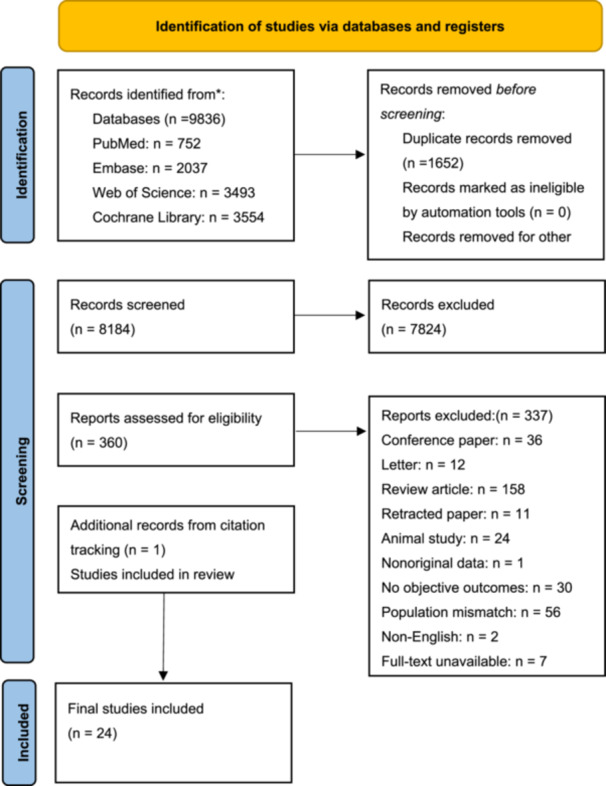
Preferred reporting items for systematic reviews and meta‐analyses flow diagram showing the review search strategy.

The final literature search identified 9836 references. After 1652 duplicates were excluded, 8184 studies were screened for title and abstract, resulting in 337 full texts. One additional study was included through citation tracking (*n* = 1), and a total of 24 studies [15, 16, 17, 18, 19, 20, 21, 22, 23, 24, 25, 26, 27, 28, 29, 30, 31, 32, 33, 34, 35, 36, 37, 38] were ultimately included (Figure 1). Current evidence regarding the explanatory variables of 24 h activity behaviors in patients with coronary heart disease remains limited. Seven studies jointly examined the determinants of both physical activity and sedentary behavior in coronary heart disease patients; 13 studies focused solely on physical activity, one study investigated only sedentary behavior, and three studies addressed sleep duration. No study has systematically explored the explanatory variables for all three activity behaviors. Moreover, the objective measurement tools used across these studies varied, with accelerometers being the most commonly employed device. More details on the study characteristics can be found in Supporting Information S1: File 2.

### 3.2 Quality Assessment Results

Among the nine cohort studies, two repeated‐measures studies, two prospective studies, and four longitudinal studies, seven were classified as high‐quality studies [17, 18, 19, 21, 22, 24, 31] (The Newcastle–Ottawa Scale score ≥ 7), seven as moderate‐quality studies [16, 20, 23, 25, 26, 29, 32] (The Newcastle–Ottawa Scales core of 6), and three as low‐quality studies [27, 28, 30] (The Newcastle–Ottawa Scale score ≤ 5). Among the seven cross‐sectional studies, four exhibited a low risk of bias [33, 34, 35, 36], two had a moderate risk of bias [15, 37], and one had a high risk of bias [38]. Notably, Item 5 (“Identification of Confounding Factors”) and Item 6 (“Strategies for Managing Confounding Factors”) had the highest frequency of “not reported” responses across all the assessments. Detailed methodological evaluation results, including specific domain scores, are provided in Supporting Information S1: File 3.

## 4 Explanatory Variables of 24 h Movement Behaviors in People With Coronary Heart Disease

**Figure 2:**
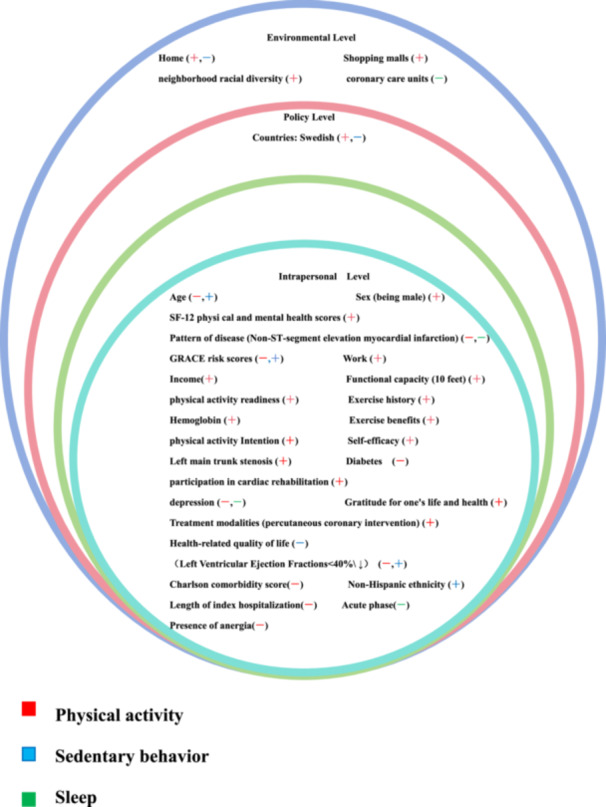
Explanatory variables for physical activity, sedentary behavior, and sleep in coronary heart disease patients according to the four domains of the socioecological model. *Note:* Physical activity: Also referred to as “body activity,” physical activity is defined as physiological movement based on skeletal muscle contraction that results in displacement and sustained energy expenditure. Based on intensity, physical activity is categorized into light, moderate, and moderate‐to‐vigorous intensities. In this review, physical activity was operationalized as the duration of activity or accumulated steps, as quantified by objective monitoring devices (e.g., accelerometers). Sedentary behavior: Refers to static activities performed in a sitting, reclining, or lying posture while awake, characterized by a low energy expenditure (≤ 1.5 metabolic equivalents). In this review, sedentary behavior was operationalized as total sedentary time, measured objectively via monitoring devices. Sleep: Defined as a periodic state of rest during which conscious activity temporarily ceases. In this review, sleep was operationalized as sleep duration, uniformly recorded by objective monitoring devices. “0” indicated no association (0%–33% of studies support the hypothesized association), “?” indicated unclear conclusions (34%–59% of studies supported the association), and “+” or “–” indicated a positive or negative correlation (≥ 60% of studies supported the direction of the relationship).

Despite extensive research on individual activity behaviors, no study has systematically examined the factors influencing 24 h activity behaviors in patients with coronary heart disease. Thus, the present systematic review synthesizes the explanatory variables for physical activity, sedentary behavior, and sleep. As shown in Figure 2 and Table 1, these influencing factors were systematically categorized according to the multilevel framework of the socioecological model, creating a structured system of determinants across four domains: individual, interpersonal, environmental, and policy. Together, these factors shape the 24 h movement behavior patterns of the coronary heart disease population.

**Table 1:** Explanatory variables for physical activity, sedentary behavior, and sleep in coronary heart disease patients and strength of association.

	Related to physical activity		Unrelated to physical activity	Summary codea	
	Positive association	Negative association		*n*/*N* for row	Association (+/−/0/?)
Intrapersonal level					
Age		H. Zetterberg 2025 (15), Lönn A 2024 (16), Goodwin A Metal 2019 (25), Green P 2013 (24), M. Moghe 2019 (31)		5/5 (100%)	−
Sex (being male)	Goodwin A Metal 2019 (25), M. Moghe 2019 (31)			2/2 (100%)	+
SF‐12 physical and mental health scores	Goodwin A Metal 2019 (25)			1/1 (100%)	+
Pattern of disease (being non‐ST‐segment elevation myocardial infarction)		S. J. Charman 2023 (27)		1/1 (100%)	−
GRACE risk scores		Green P2013 (24)		1/1 (100%)	−
Work	M. Moghe 2019 (31), Zetterberg 2025 (15)			2/2 (100%)	+
Income	M. Moghe 2019 (31)			1/1 (100%)	+
Functional capacity (10 feet)	Byun W 2014 (33)			1/1 (100%)	+
Physical activity readiness	Byun W 2014 (33), M. Moghe 2019 (31)			2/2 (100%)	+
Exercise history	Byun W 2014 (33)			1/1 (100%)	+
Hemoglobin	Kuya F 2023 (18)			1/1 (100%)	+
Exercise benefits	M. Moghe 2019 (31)			1/1 (100%)	+
Physical activity intention	M. Moghe 2019 (31), H. Zetterberg 2025 (15)			2/2 (100%)	+
Self‐efficacy	M. Moghe 2019 (31), Kuya F 2023 (18)		Shajrawi A 2020 (29)	2/3 (66.7%)	+
Left main trunk stenosis	Kuya F 2023 (18)			1/1 (100%)	+
Indices of greater disease severity (left ventricular ejection fraction ↓\diabetes\Charlson comorbidity score\length of index hospitalization\the presence of anergia)		Kuya F 2023 (18), H. Zetterberg 2025 (15), Green P 2013 (24)		3/3 (100%)	−
Participation in cardiac rehabilitation	Kuya F 2023 (18), N. Freene 2020 (19), Ter Hoeve N 2017 (22), Ramadi A 2016 (26), Stevenson TG (37), Jones NL 2007 (38)			6/6 (100%)	+
Depression		Kuya F 2023 (18)		1/1 (100%)	−
Gratitude for one's life and health	Legler SR 2019 (32)			1/1 (100%)	+
Treatment modalities (being percutaneous coronary intervention\treatment with percutaneous transtuminal coronary angioplasty during acute coronary syndrome hospitalization)	Shajrawi A 2019 (30), Green P 2013 (24)			2/2 (100%)	+
Interpersonal level					
Spouse\social support	Green P 2013 (24), H. Zetterberg 2025 (15)			2/2 (100%)	+
Environmental level					
Home	Trecarten N 2021 (28), B. M. A. van Bakel 2023 (17)			2/2 (100%)	+
Countries (Swedish)	N. Freene 2020 (20)			1/1 (100%)	+
Shopping malls	M. Moghe 2019 (31)			1/1 (100%)	+
Neighborhood racial diversity	Ellen‐ge Denton 2014 (36)			1/1 (100%)	+
Policy level					
Cardiac rehabilitation delivery models			Ramadi A 2016 (26)	0/1 (100%)	0

	Related to sedentary behavior		Unrelated to sedentary behavior	Summary codea	
	Positive association	Negative association		*n*/*N* for row	Association (+/−/0/?)
Intrapersonal level					
Age	Lönn A 2024 (16)				
Health‐related quality of life		Andrea T Duran 2019 (21)		1/1 (100%)	−
Indices of greater disease severity (left ventricular ejection fraction < 40%\GRACE risk scores)	Andrea T Duran 2019 (21)			1/1 (100%)	+
Non‐Hispanic ethnicity	Andrea T Duran 2019 (21)			1/1 (100%)	+
Interpersonal level					
Spouse		Andrea T Duran 2019 (21)		1/1 (100%)	−
Environmental level					
Home		B. M. A. van Bakel 2023 (17)	Trecarten N 2021 (28)	1/2 (50%)	?
Policy level					
Countries (Swedish)		N. Freene 2020 (20)		1/1 (100%)	−

	Related to sleep		Unrelated to sleep	Summary codea	
	Positive association	Negative association		*n*/*N* for row	Association (+/−/0/?)
Intrapersonal level					
Emotional distress		Ulla M Edéll‐Gustaffson 2002 (36)		1/1 (100%)	−
Pattern of disease (being non‐ST‐segment elevation myocardial infarction)		S. J. Charman 2023 (27)		1/1 (100%)	−
Acute phase		Schiza SE 2010 (35)		1/1 (100%)	−
Environmental level					
Coronary care units		Hadil Al Otair (34)		1/1 (100%)	−

### 4.1 Intrapersonal Level

#### 4.1.1 Sociodemographic Characteristics

Evidence consistently indicates that advanced age is associated with reduced physical activity [15, 16, 24, 25, 31] (5/5 studies, 100%) and increased sedentary behavior [16] (1/1 study, 100%). Male sex correlates with higher physical activity levels [25, 31] (2/2 studies, 100%). Employment status is positively correlated with increased physical activity, and for women, both work status and annual income are associated with physical activity volume [15, 31] (2/2 studies, 100%).

#### 4.1.2 Psychological Factors

The association between self‐efficacy and physical activity is heterogeneous [18, 29, 32] (2/3 studies, 66.7%). Patients with poor exercise habits consistently exhibit lower physical activity levels [30] (1/1 study, 100%), whereas a disposition toward gratitude predicts higher physical activity participation [32] (1/1 study, 100%). Conversely, anxiety and depression are linked to reduced sleep duration [37] (1/1 study, 100%).

#### 4.1.3 Clinical Indicators

Greater disease severity (e.g., reduced left ventricular ejection fraction, higher comorbidity burden) consistently predicts lower physical activity [15, 21] (2/2 studies, 100%). Conversely, higher functional capacity (e.g., exercise tolerance) is associated with greater physical activity engagement [15, 21, 33] (3/3 studies, 100%), as are elevated hemoglobin levels [15] (1/1 study, 100%). The acute phase of illness significantly shortens sleep duration [35] (1/1 study, 100%). Treatment modality also influences activity; patients undergoing direct percutaneous coronary intervention demonstrate significantly higher daily step counts during the 2–6‐week post‐discharge rehabilitation period compared to those receiving alternative treatments [30] (1/1 study, 100%). Finally, non‐ST‐elevation myocardial infarction is significantly associated with decreased physical activity and increased sedentary time [27] (1/1 study, 100%), while left main trunk stenosis appears to be a positive correlate of moderate‐to‐vigorous physical activity [18] (1/1 study, 100%).

### 4.2 Interpersonal Level

Social support, specifically cohabiting with a partner, is a key protective factor. Patients without a partner exhibited a significant 24.4% reduction in daytime physical activity during the first month post‐discharge compared to those with partners [24] (1/1 study, 100%). Correspondingly, the absence of a partner also predicts significantly longer total sedentary time [21] (1/1 study, 100%).

### 4.3 Environmental Level

#### 4.3.1 Residential Environment

The home environment demonstrates greater potential for promoting physical activity compared to restrictive hospital settings [17, 28] (2/2 studies, 100%). Excessive environmental stimuli in coronary care units, such as continuous equipment noise and constant artificial lighting, disrupt circadian rhythms, leading to fragmented sleep [34] (1/1 study, 100%). At the community level, ethnically diverse neighborhoods leveraging cultural heterogeneity to develop accessible physical activity infrastructure systematically enhance group activity participation [23] (1/1 study, 100%).

#### 4.3.2 Policy and Healthcare Resource Allocation Background

Participation in cardiac rehabilitation emerges as the most robust predictor of increased physical activity (6/6 studies, 100%) [18, 19, 22, 26, 37, 38]. Disparities exist between healthcare systems; patients in Sweden exhibit higher physical activity levels than those in Australia [20] (1/1 study, 100%), likely reflecting differences in post‐intervention inpatient education and pre‐existing population activity levels. It should be noted that neither traditional cardiac rehabilitation models nor accelerated exercise programs have been conclusively shown to achieve sustained long‐term changes in physical activity behavior [26] (1/1 study, 100%).

## 5 Discussion

The most of investigated determinants are concentrated at the individual level (e.g., age, sex, physical health status), while exploration at the interpersonal and environmental levels remains underdeveloped. This pattern reflects a systemic deficiency in the application of socioecological methodologies within the existing research framework. The most of investigated determinants are concentrated at the individual level (e.g., age, sex, physical health status), while exploration at the interpersonal and environmental levels remains underdeveloped. This pattern reflects a systemic deficiency in the application of socioecological methodologies within the existing research framework. By focusing on interactions across social‐ecological levels, behavior change interventions have shown positive effects in healthy populations [39], however, their application in coronary heart disease populations remains underexplored. Future research should apply socioecological model to explore the factors influencing 24 h movement behaviors in coronary heart disease patients, providing guidance for future intervention studies.

At the intrapersonal level, advanced age, female sex, and poorer physical health are consistently associated with reduced physical activity and increased sedentary time, which mirrors patterns observed in the general population [40, 41]. Given that age and sex are non‐modifiable biological markers, their clinical utility lies in risk stratification rather than causal intervention. For instance, recognizing that elderly female patients face a heightened risk of inactivity should prompt clinicians to allocate targeted resources to this subgroup.

At the interpersonal level, the absence of a partner emerges as a social risk factor for decreased physical activity and increased sedentary time post‐discharge, aligning with findings from general adults [42, 43]. Unlike fixed demographic markers, social support represents a reversible psychosocial process. For patients lacking a partner (regardless of marital status history), establishing “clinician−patient partnerships” or peer support groups can effectively substitute for the supervisory role of a spouse, thereby mitigating the risk of inactivity.

At the environmental and policy level, sedentary time is significantly longer in hospital settings than in home‐based contexts. This discrepancy is largely attributable to institutional constraints, including standardized diagnostic and treatment workflows and mandatory bed rest orders, which passively increase patients' overall sedentary exposure. Accordingly, early ambulation tailored to individual disease severity should be prioritized to reduce unnecessary bed rest and mitigate avoidable sedentary accumulation during hospitalization. Findings from community‐level research on culturally adapted physical activity facilities further indicate that environmental interventions should not focus exclusively on hardware infrastructure. Instead, such interventions must be aligned with the cultural characteristics and usage preferences of target populations to effectively improve facility utilization rates and population‐level physical activity participation.

The short‐term efficacy of cardiac rehabilitation in promoting physical activity has been validated across multiple independent studies. Nevertheless, conventional cardiac rehabilitation programs consistently fail to support long‐term maintenance of behavior change, representing a pervasive and longstanding challenge in global cardiac rehabilitation practice. To address this gap, an integrated three‐tier digital linkage model spanning hospital rehabilitation, community support, and home‐based maintenance is proposed. Specifically, hospital teams assume responsibility for acute‐phase clinical stabilization and individualized exercise prescription; community health providers deliver transitional supervision and foster peer social support during the recovery period; and wearable devices paired with remote monitoring platforms extend standardized rehabilitation guidance seamlessly into the home setting. Furthermore, cross‐national disparities in patient activity patterns corroborate the formative influence of healthcare systems and rehabilitation policies on health behaviors among patients with coronary heart disease. This evidence underscores the necessity of context‐specific adaptation of intervention strategies to match local healthcare infrastructures and population characteristics, rather than direct unadapted translation of foreign models.

The beneficial effects of increasing physical activity and reducing sedentary behavior have been well documented in healthy populations. However, in‐depth exploration of sleep‐related behaviors remains insufficient, and empirical evidence on the determinants of objectively measured sleep duration is particularly scarce. Existing evidence demonstrates a U‐shaped association between sleep duration and the risks of coronary heart disease incidence and cardiovascular mortality, with a stronger magnitude of effect observed for mortality risk relative to incidence risk [44]. Accordingly, additional empirical research focusing on the determinants of sleep is warranted to comprehensively elucidate its underlying pathophysiological mechanisms and inform the development of targeted precision interventions.

## 6 Conclusion

On the basis of the socioecological model, this study systematically examined the multilevel factors influencing 24 h movement behaviors in patients with coronary heart disease. Future research should adopt longitudinal designs to clarify the temporal and causal relationships between socioecological variables and the evolution of these behaviors, thereby contributing to the establishment of an integrated policy support network that links healthcare, community, and family systems. Since the health benefits of time‐use patterns in 24 h activities exhibit dose–response relationships, further evidence is needed to better understand the socioecological determinants of these behaviors in coronary heart disease patients. Such evidence will be crucial for the development of comprehensive 24 h activity management protocols and optimization of whole‐cycle health management for patients with coronary heart disease.

## 7 Strengths and Limitations

This study included only research that employed objective measurement methods, minimizing the risk of recall bias and the over‐ or underestimation of activity behaviors associated with subjective measurement techniques [45, 46]. The majority of the studies included in this review exhibited a low risk of bias and high quality. However, several limitations were identified. One key limitation was the high heterogeneity in measurement concepts, particularly the significant variability in the objective measurement indicators for physical activity, sedentary behavior, and sleep. This variability may hinder meaningful comparisons between behavior categories. Additionally, data on explanatory variables may have been missing, as the exclusion of self‐reported studies may have led to the omission of valuable evidence on these factors. Furthermore, the evidence base remains relatively weak, as the range of explanatory variables investigated was both broad and dispersed. Some variables were explored in only one study, and only seven studies simultaneously examined more than two types of activity behaviors. Additionally, research on the integration of 24 h movement behavior patterns was lacking. Finally, most primary studies included in this review adopted a cross‐sectional design without multivariate adjustment for confounding factors. Accordingly, the directions of associations summarized in this review may be substantially biased by the analytical approaches of original studies. In addition, these studies failed to account for cases where confidence intervals contained zero values, which raises the risk of falsely interpreting random measurement error as genuine associations. Longitudinal studies are necessary to confirm the present findings. Future research should focus on multicenter longitudinal cohort studies to address these gaps.

## Author Contributions

X.F. and H.T. were responsible for the study design. X.F. performed the data analysis and drafted the manuscript. X.Z. and Y.W. assisted with the data analysis. T.G. and S.Y. contributed to the interpretation of the results. T.C. and Y.P. were responsible for editing the manuscript. All authors read and approved the final version of the manuscript.

## Conflicts of Interest

The authors declare no conflicts of interest.

## Supporting information


Supporting File

## Data Availability

The data that support the findings of this study are available from the corresponding author upon reasonable request.
