# Supplementary material for: Explanatory Variables of Objectively Measured 24 h Movement Behaviors in Coronary Heart Disease Patients: A Systematic Review
Source: Clin Cardiol. 2026 Aug 17;49(8):e70440. doi: 10.1002/clc.70440 (PMC13479476; doi:10.1002/clc.70440)
Supplement: Supplementary file 1 [file CLC-49-e70440-s001.docx]

Supplementary File 1

Pubmed752

(((Accelerometry[MeSH Terms]) OR (Accelerometry[TIAB]) OR (Accelerometer[TIAB]) OR (accelerometers[TIAB]) OR (accelerometer-derived[TIAB]) OR (Motion Sensor[TIAB]) OR (Motion sensors[TIAB]) OR (objective evidence[TIAB]) OR (objective assessment[TIAB]) OR (objective approach[TIAB]) OR (objective measure[TIAB]) OR (Objective measurement[TIAB]) OR (Objective evaluation[TIAB]) OR (Objective manner[TIAB]) OR (Objective data[TIAB]) OR (Objective research[TIAB]) OR (Objective factor[TIAB]) OR (Objective method[TIAB]) OR (objective description[TIAB]) OR (objective reporting[TIAB]) OR (objective observation[TIAB]) OR (objective result[TIAB]) OR (objective finding[TIAB]) OR (objective investigation[TIAB]) OR (objective outcome[TIAB]) OR (objective instrument[TIAB]) OR (objective information[TIAB]) OR (objectively[TIAB]) OR (device measured[TIAB]) OR (wearable[TIAB]) OR (wearables[TIAB]) OR (activity tracker[TIAB]) OR (activity trackers[TIAB]) OR (activity monitor[TIAB]) OR (activity monitors[TIAB]) OR (multisensory-device[TIAB]) OR (multisensory-devices[TIAB]) OR (multisensory device[TIAB]) OR (multisensory devices[TIAB]) OR (monitoring app[TIAB]) OR (monitoring apps[TIAB]) OR (monitoring application[TIAB]) OR (Monitoring applications[TIAB]) OR (monitoring system[TIAB]) OR (polysomnography[TIAB]) OR (PSG[TIAB]) OR (bed sensor[TIAB]) OR (sleep sensor[TIAB]) OR (eyelid movement[TIAB]) OR (sleep switch[TIAB]) OR (remote device[TIAB]) OR (sleep timer[TIAB]) OR (sleep timing[TIAB]) OR (Apple watch[TIAB]) OR (Apple watches[TIAB]) OR (Sensewear[TIAB]) OR (Actigraphy[MeSH Terms]) OR (Actigraphy[TIAB]) OR (Actigraph[TIAB]) OR (Actigraphs[TIAB])) AND ((Exercise[MeSH Terms]) OR (Exercise[Title/Abstract]) OR (Physical activity[Title/Abstract]) OR (Physical activities[Title/Abstract]) OR (walking[MeSH Terms]) OR (walking[Title/Abstract]) OR (walk[Title/Abstract]) OR (Running[MeSH Terms]) OR (Running[Title/Abstract]) OR (Run[Title/Abstract]) OR (Leisure activity[Title/Abstract]) OR (Leisure Activities[MeSH Terms]) OR (Leisure Activities[Title/Abstract]) OR (sport[Title/Abstract]) OR (Sports[MeSH Terms]) OR (Sports[Title/Abstract]) OR (motor activity[Title/Abstract]) OR (motor activities[Title/Abstract]) OR (Locomotor activity[Title/Abstract]) OR (locomotor activities[Title/Abstract]) OR (Physical movement[Title/Abstract]) OR (Physical movements[Title/Abstract]) OR (Physical performance[Title/Abstract]) OR (physical performances[Title/Abstract]) OR (Physical effort[Title/Abstract]) OR (Physical efforts[Title/Abstract]) OR (Break[Title/Abstract]) OR (Breaks[Title/Abstract]) OR (Sedentary Behaviour[Title/Abstract]) OR (Sedentary Behaviours[Title/Abstract]) OR (Sedentary Behavior[MeSH Terms]) OR (Sedentary Behavior[Title/Abstract]) OR (Sedentary Behaviours[Title/Abstract]) OR (Sedentary[Title/Abstract]) OR (Sedentariness[Title/Abstract]) OR (Physical inactivity[Title/Abstract]) OR (Lack of physical activity[Title/Abstract]) OR (sitting[Title/Abstract]) OR (sit[Title/Abstract]) OR (Stationary[Title/Abstract]) OR (active transport[Title/Abstract]) OR (passive transport[Title/Abstract]) OR (Motor transport[Title/Abstract]) OR (Screen time[Title/Abstract]) OR (Screen use[Title/Abstract]) OR (Television[Title/Abstract]) OR (TV[Title/Abstract]) OR (Tablet[Title/Abstract]) OR (Smartphone[Title/Abstract]) OR (Reading[Title/Abstract]) OR (sleep[MeSH Terms]) OR (sleep[Title/Abstract]) OR (sleeping[Title/Abstract]) OR (lying[Title/Abstract]) OR (Nap[Title/Abstract]) OR (Napping[Title/Abstract]) OR (sleepiness) OR (24h movement[Title/Abstract]) OR (24h movements[Title/Abstract]) OR (24 hours movement[Title/Abstract]) OR (24 hours movements[Title/Abstract]) OR (24h use of time[Title/Abstract]) OR (24h day[Title/Abstract]))) AND ((Coronary Artery Disease[MeSH Terms]) OR (Coronary Artery Diseases[Title/Abstract]) OR (Coronary Arteriosclerosis[Title/Abstract]) OR (Coronary Atheroscleroses[Title/Abstract]) OR (Coronary Atherosclerosis[Title/Abstract]) OR (Left Main Coronary Artery Disease[Title/Abstract]) OR (Left Main Coronary Disease[Title/Abstract]) OR (Left Main Disease[Title/Abstract]) OR (Left Main Diseases[Title/Abstract]) OR (Coronary Disease[MeSH Terms]) OR (Coronary Diseases[Title/Abstract]) OR (Coronary Heart Disease[Title/Abstract]) OR (Coronary Heart Diseases[Title/Abstract]) OR (acute myocardial infarction[Title/Abstract]) OR (AMl[Title/Abstract]) OR (acute coronary syndrome[Title/Abstract]) OR (angina[Title/Abstract]))

Web of science 3493

TS=(“Coronary Artery Disease” OR “Coronary Artery Diseases” OR “Coronary Arteriosclerosis” OR “Coronary Atheroscleroses” OR “Coronary Atherosclerosis” OR “Left Main Coronary Artery Disease” OR “Left Main Coronary Disease” OR “Left Main Disease” OR “Left Main Diseases OR Coronary Disease” OR “Coronary Diseases” OR “Coronary Heart Disease” OR “Coronary Heart Diseases” OR “acute myocardial infarction” OR “AMl” OR “acute coronary syndrome” OR “angina”)1

**TS=(“24h movement” OR “24h movements” OR “24 hours movement” OR “24 hours movements” OR “24h use of time” OR “24h day”OR “24h movement” OR “24h movements” OR “24 hours movement” OR “24 hours movements” OR “24h use of time” OR “24h day” OR “Exercise” OR “Physical activity” OR “Physical activities” OR “walking” OR “walk” OR “Running” OR “Run” OR “Leisure activity” OR “Leisure Activities” OR “sport” OR “Sports” OR “motor activity” OR “motor activities” OR “Locomotor activity” OR “locomotor activities” OR “Physical movement” OR “Physical movements” OR “Physical performance” OR “physical performances” OR “Physical effort” OR “Physical efforts” OR “Break” OR “Breaks” OR “Sedentary Behavior” OR “Sedentary Behaviors” OR “Sedentary Behaviour” OR “Sedentary Behaviours” OR “Sedentary” OR “Sedentariness” OR “Physical inactivity” OR “Lack of physical activity” OR “sitting” OR “sit” OR “Stationary” OR “active transport” OR “passive transport” OR “Motor transport” OR “Screen time” OR “Screen use” OR “Smartphone” OR “Reading” OR “sleep” OR “sleeping” OR “lying” OR “sleepiness”)2**

TS=(“Accelerometry” OR “Accelerometer” OR “accelerometers” OR “accelerometer-derived” OR “Motion Sensor” OR “Motion sensors” OR “observation” OR “observations” OR “objectively” OR “device measured” OR “wearable” OR “wearables” OR “activity tracker” OR “activity trackers” OR “activity monitor” OR “activity monitors” OR “multisensory-device” OR “multisensory-devices” OR “multisensory device” OR “multisensory devices” OR “polysomnography” OR “PSG” OR “bed sensor” OR “sleep sensor” OR “eyelid movement” OR “sleep switch” OR “remote device” OR “sleep timer” OR “sleep timing” OR “Apple watch” OR “Apple watches” OR “Sensewear” OR “Actigraphy” OR “Actigraph” OR “Actigraphs”）3

1 AND 2AND 3

Embase2037

1

'coronary artery disease'/exp OR 'coronary disease':ab,ti OR 'multivessel coronary artery disease':ab,ti OR 'coronary artery disease':ab,ti OR 'coronary atheroscleroses':ab,ti OR 'coronary atherosclerosis':ab,ti OR 'left main coronary artery disease':ab,ti OR 'left main coronary disease':ab,ti OR 'left main disease':ab,ti OR 'left main diseases or coronary disease':ab,ti OR 'coronary diseases':ab,ti OR 'coronary heart disease':ab,ti OR 'coronary heart diseases':ab,ti OR 'acute myocardial infarction':ab,ti OR 'aml':ab,ti OR 'acute coronary syndrome':ab,ti OR 'angina':ab,ti

2

'physical activity'/exp OR 'activity, physica':ab,ti OR 'physical activity':ab,ti OR 'sedentary lifestyle'/exp OR 'sedentary behavior':ab,ti OR 'sedentary behaviour':ab,ti OR 'sedentary life style':ab,ti OR 'sedentary lifestyle':ab,ti OR 'sleep'/exp OR sleeping:ab,ti OR sleep:ab,ti OR 'sitting'/exp OR 'activity, sedentary':ab,ti OR 'posture, sitting':ab,ti OR 'seated position':ab,ti OR 'sedentary activity':ab,ti OR 'sedentary work':ab,ti OR 'sitting position':ab,ti OR 'sitting posture':ab,ti OR sitting:ab,ti OR 'screen time'/exp OR 'screen-use time':ab,ti OR 'screen-viewing time':ab,ti OR 'screen-watching time':ab,ti OR screentime:ab,ti OR 'screen time':ab,ti OR '24h movement':ab,ti OR '24h movements':ab,ti OR '24 hours movement':ab,ti OR '24 hours movements':ab,ti OR '24h use of time':ab,ti OR '24h day':ab,ti

3

'accelerometry'/exp OR 'accelerometer':ab,ti OR 'accelerometers':ab,ti OR 'accelerometer-derived':ab,ti OR 'motion sensor':ab,ti OR 'motion sensors':ab,ti OR 'observation':ab,ti OR 'observations':ab,ti OR 'objectively':ab,ti OR 'device measured':ab,ti OR 'wearable':ab,ti OR 'wearables':ab,ti OR 'activity tracker':ab,ti OR 'activity trackers':ab,ti OR 'activity monitor':ab,ti OR 'activity monitors':ab,ti OR 'multisensory-device':ab,ti OR 'multisensory-devices':ab,ti OR 'multisensory device':ab,ti OR 'multisensory devices':ab,ti OR 'polysomnography':ab,ti OR 'psg':ab,ti OR 'bed sensor':ab,ti OR 'sleep sensor':ab,ti OR 'eyelid movement':ab,ti OR 'sleep switch':ab,ti OR 'remote device':ab,ti OR 'sleep timer':ab,ti OR 'sleep timing':ab,ti OR 'apple watch':ab,ti OR 'apple watches':ab,ti OR 'sensewear':ab,ti OR 'actigraphy':ab,ti OR 'actigraph':ab,ti OR 'actigraphs':ab,ti

#1 AND #2 AND #3

Cochrane3554

Coronary Disease(MeSH)1

Coronary Heart Diseases; Coronary Diseases; Disease, Coronary; Heart Diseases, Coronary; Coronary Heart Disease; Diseases, Coronary Heart; Heart Disease, Coronary; Diseases, Coronary; Disease, Coronary Heart2

1OR 2

Exercise（MeSH）3

Activities, Physical; Physical Activities; Activity, Physical; Physical Activity; Exercises, Isometric; Exercise, Isometric; Isometric Exercises; Isometric Exercise; Exercise Trainings; Exercise Training; Trainings, Exercise; Training, Exercise; Exercise, Aerobic; Aerobic Exercise; Aerobic Exercises; Exercises, Aerobic; Acute Exercises; Acute Exercise; Exercise, Acute; Exercises, Acute; Physical Exercises; Exercise, Physical; Exercises, Physical; Exercises; Physical Exercise5

Sedentary Behavior（MeSH）6

Lack of Physical Activity; Inactivity, Physical; Physical Inactivity; Sedentary Behaviors; Lifestyle, Sedentary; Behavior, Sedentary; Sedentary Lifestyle; Sedentary Times; Sedentary Time; Time, Sedentary

Sit ;Sitting ;Screen Times7

Screen Time（MeSH）8

24h movement OR 24h movements OR 24 hours movement OR 24 hours movements OR 24h use of time OR 24h day9

Accelerometry（MeSH）10

Accelerometry OR Accelerometer OR accelerometers OR accelerometer-derived OR Motion Sensor OR Motion sensors OR observation OR observations OR objectively OR device measured OR wearable OR wearables OR activity tracker OR activity trackers OR activity monitor OR activity monitors OR multisensory-device OR multisensory-devices OR multisensory device OR multisensory devices OR polysomnography OR PSG OR bed sensor OR sleep sensor OR eyelid movement OR sleep switch OR remote device OR sleep timer OR sleep timing OR Apple watch OR Apple watches OR Sensewear OR Actigraphy OR Actigraph OR Actigraphs11

Supplementary File 2

| **Author** | **population** | **year** | **State** | **Research subjects** | **Sample size** | **Objective equipment** | **Influencing factors** | **outcome** |
| --- | --- | --- | --- | --- | --- | --- | --- | --- |
| Hedvig Zetterberg | Cross-sectional study | 2025 | Sweden | ACS | 100 | （Actigraph wGT3X-BT\GT9X\ActiGraph LCC） | Age work LVEF↓ social support levels,  expected outcomes of physical activity (PA)  PA motivtion. | PA |
| Amanda Lönn | Prospective cohort study | 2024 | Sweden | MI | 178 | Actigraph GT3X | Age | PA\SB |
| Charman, Sarah J. | Longitudinal observational study | 2023 | UK | ACS \ stable angina pectoris elective | 58 | GENEActiv | pattern of disease (being Non-ST-segment elevation myocardial infarction ) | MVPA\LAP\SP |
| B. M. A. van Bakel | Prospective cohort study | 2023 | Netherlands | MI | 165 | ActivPAL3 micro | home | PA\SB |
| Neil Trecarten | Cross-sectional study Longitudinal observational study | 2021 | Canada | CAD | 318 | GT3X (Actigraph) accelerometer | home | PA\SB |
| N. Freene | Prospective cohort study | 2020 | Australia | CAD | 72 | ActiGraph ActiSleep | participation in cardiac rehabilitation | PA\SB |
| N. Freene | Prospective cohort study | 2020 | Australia | PCI | 183(Australia n = 50， Sweden n = 133) | ActiGraph ActiSleep | Countries（Swedish） | PA\SB |
| Shajrawi.A | Longitudinal study with repeated measures design | 2020 | Jordan | AMI | 100 | activPAL3 | Self-Efficacy | PA |
| A. Shajraw | Repeated measures design | 2019 | Jordan | AMI | 94 | activPAL3 monitor | treatment modalities (being PCI) | PA |
| M. Moghe | Cross-sectional study Longitudinal observational study | 2019 | Canada | CAD | 449 | Actigraph GT3X+ | Age work PA readiness  Exercise Benefits PA Intention  Self-Efficacy shopping malls | MVPA |
| S. R. Legler | Prospective study | 2019 | USA | ACS | 152 | Fitlinxx Pebble accelerometer | gratitude for one's life and health | PA |
| Ashley M Goodwin | Observational cohort study | 2019 | USA | ACS | 151 | Actical accelerometer | sex age SF-12 physical and mental health scores | PA |
| A. T. Duran | Observational cohort study | 2019 | USA | ACS | 149 | Actical accelerometer | LVEF <40% GRACE risk scores Spouse health-related quality of life Non-Hispanic ethnicity | SB |
| Nienke Ter Hoeve | Prospective cohort study | 2017 | Netherlands | ACS | 135 | Actigraph GT3X+ | participation in cardiac rehabilitation | MVPA\SB |
| Ailar Ramadi | Prospective repeated-measures study | 2016 | Canada | CAD | 44 | Sense Wear Mini Armband | cardiac rehabilitation delivery models | PA |
| Ellen-ge Denton | Prospective study | 2014 | USA | ACS | 87 | continuous activity monitors，Actical® （Philips – Respironics， Inc， Bend， Oregon） | neighborhood racial diversity | PA |
| Byun.W | Cross-sectional study | 2014 | USA | CAD | 65 | Actigraph GT3X | Functional capacity(10 feet) PA readiness exercise history | PA |
| Green. P | Prospective cohort study | 2013 | USA | ACS | 107 | Actical accelerometers | Spouse  age  Charlson comorbidity score the presence of anergia (low energy) history of stroke   treatment with PTCA during ACS hospitalization and length of index hospitalization | PA |
| Hadil Al Otair | Cross-sectional observational study | 2011 | Saudi Arabia | ACS | 46 | SenseWear Pro Armband™ | CCU | SP |
| Sophia E. Schiza | Cross-sectional observational study | 2010 | Greece | ACS | 22 | Polysomnography | acute phase | SP |
| Stevenson, Troy G | Cross-sectional observational study | 2009 | USA | PCI\CABG | 22 | ActiGraph GT1M  accelerometers | participation in cardiac rehabilitation | PA |
| Jones NL | Cross-sectional observational study | 2007 | USA | CAD | 25 | ActiGraph GT1M  accelerometers | participation in cardiac rehabilitation | PA |
| Ulla M Edéll-Gustaffson | Cross-sectional study | 2002 | Sweden | CAD | 44 | Polysomnographic | emotional distress | SP |
| [Kuya Funaki](https://pubmed.ncbi.nlm.nih.gov/?sort=date&term=Funaki+K&cauthor_id=36812917) | Prospective cohort study | 2023 | Japan | PCI | 577 | Kenz Lifecorder | participation in cardiac rehabilitation depression Left main trunk stenosis diabetes Hemoglobin SeIf-Efficacy | PA |
| moderate-to-vigorous physical activity (MVPA), physical activity (PA), sedentary behavior (SB), and sleep (SP) | | | | | | | | |

Supplementary File 3

| The Newcastle–Ottawa Scale | | | | | |
| --- | --- | --- | --- | --- | --- |
| study | selection of study population | comparability between groups | outcome assessment | score | outcome |
| 2024Amanda Lönn | 3 | 2 | 2 | 7 | Medium quality |
| 2023 Bakel | 4 | 2 | 2 | 8 | high quality |
| 2023Kuya Funaki | 3 | 2 | 2 | 7 | high quality |
| 2023Charman, Sarah J | 2 | 1 |  | 5 | poor quality |
| 2021Neil Trecarten | 2 | 1 | 2 | 5 | poor quality |
| 2020N. Freene | 4 | 2 | 2 | 8 | high quality |
| 2020N. Freene | 3 | 1 | 2 | 6 | Medium quality |
| 2019 Ashley M Goodwin | 3 | 1 | 2 | 6 | Medium quality |
| 2019 A.T.Duran | 4 | 2 | 2 | 8 | high quality |
| 2020 Shajrawi | 3 | 1 | 2 | 6 | Medium quality |
| 2019 Shajrawi | 2 | 1 | 1 | 5 | poor quality |
| 2019 M. Moghe | 4 | 2 | 2 | 8 | high quality |
| 2019.S R Legler | 3 | 2 | 3 | 8 | Medium quality |
| 2017 Nienke Ter Hoeve | 4 | 1 | 3 | 8 | high quality |
| 2016 Ailar Ramadi | 3 | 1 | 2 | 6 | Medium quality |
| 2014 Ellen-ge Denton | 3 | 1 | 2 | 6 | Medium quality |
| 2013 Philip Green | 3 | 2 | 2 | 7 | high quality |

| Joanna Briggs Institute (JBI) Critical Appraisal Checklist for Analytical Cross-Sectional Studies | | | | | | | | | | |
| --- | --- | --- | --- | --- | --- | --- | --- | --- | --- | --- |
| study | Were the criteria for inclusion in the sample clearly  defined? | Were the study subjects and the setting described in  detail? | Was the exposure measured in a valid and reliable  way? | Was the exposure measured in a valid and reliable  way? | Were confounding factors identified? | Were strategies to deal with confounding factors  stated? | Were the outcomes measured in a valid and reliable  way?  8. Was appropr | Was appropriate statistical analysis used? | ratio | bias risk |
| 2025Hedvig Zetterberg | yes | yes | yes | yes | no | no | yes | no | 0.625 | Moderate bias risk |
| 2014 Wonwoo Byun | yes | yes | yes | yes | no | no | yes | yes | 0.75 | Low bias risk |
| 2011 Hadil Al Otair | yes | yes | yes | yes | no | no | yes | yes | 0.75 | Low bias risk |
| 2010 Sophia E. Schiza | yes | yes | yes | yes | no | no | yes | yes | 0.75 | Low bias risk |
| 2009 Stevenson, Troy G | no | Unclear | yes | no | yes | yes | yes | no | 0.5 | Moderate bias risk |
| 2007 Jones NL | no | Unclear | yes | no | yes | no | yes | no | 0.375 | High risk of bias |
| 2002 Ulla M Edéll-Gustaffson | yes | yes | yes | yes | yes | no | yes | no | 0.75 | Low bias risk |
